# Supplementary material for: Rapid cell-free forward engineering of novel genetic ring oscillators
Source: eLife. 2015 Oct 5;4:e09771. doi: 10.7554/eLife.09771 (PMC4714972; doi:10.7554/eLife.09771)
Supplement: Supplementary file 1. — (A) Linear DNAs used in this study. (B) Plasmids used in this study. (C) Strains used in this study. (D) DNA concentrations used in experiments. DOI: http://dx.doi.org/10.7554/eLife.09771.032 [file elife-09771-supp1.zip › Supplementary file 1.pdf]

**Supplementary file 1:**

## A) Linear DNAs used in this study.

| Name | Description | Notes |
|---|---|---|
| pJ23119-tetO-BCD2-phlF-ssrA(LAA) | # 3n2, 5n1, 4n | |
| pLacI-BCD2-tetR-ssrA(LAA) | & 5n1, 4n | |
| pLambdaCI-BCD2-lacI-ssrA(LAA) | % 5n1 | |
| pPhlF-BCD2-srpR-ssrA(LAA) | # 3n2, 5n1, 4n | |
| pSprR-BCD2-lambdaCI-ssrA(LAA) | # 5n1 | |
| pSrpR-BCD2-tetR-ssrA(LAA) | # 3n2 | |
| pPhlF-BCD2-srpR | # 3n2/no-ssrA | |
| pSrpR-BCD2-tetR | # 3n2/no-ssrA | |
| pTetR-BCD2-phlF | # 3n2/no-ssrA | |
| pSrpR-BCD2-lacI-ssrA(LAA) | # 4n | |
| pBetI-BCD7-QacR-ssrA(LAA) | # 5n2 | |
| pPhlF-BCD7-srpR-ssrA(LAA) | # 5n2 | |
| pQacR-BCD7-tetR-ssrA(LAA) | # 5n2 | |
| PSrpR-BCD7-BetI-ssrA(LAA) | # 5n2 | |
| pTetR-BCD7-phlF-ssrA(LAA) | # 5n2 | |
| pJ23151-BCD7-betI | $ transfer fxns | |
| pJ23151-BCD7-lacI | $ transfer fxns | |
| pJ23151-BCD7-lambdaCI | $ transfer fxns | |
| pJ23151-BCD7-phlF | $ transfer fxns | |
| pJ23151-BCD7-qacR | $ transfer fxns | |
| pJ23151-BCD7-srpR | $ transfer fxns | |
| pJ23151-BCD7-tetR | $ transfer fxns | |
| pLacI-BCD2-sfGFP-ssrA(LAA) | & test reporter | |
| pLambdaCI-BCD2-sfGFP-ssrA(LAA) | % test reporter | |
| pPhlF-BCD2-sfGFP-ssrA(LAA) | # test reporter | |
| pSrpR-BCD2-sfGFP-ssrA(LAA) | # test reporter | |
| pTetR-BCD2-sfGFP-ssrA(LAA) | # test reporter | |

# Promoter from Stanton et al. (2014)
$ Promoter from Anderson promoter panel
% Promoter from Elowitz and Leibler (2000)
& Promoter from Lutz and Bujard (1997)

## B) Plasmids used in this study.

| Name | Description | Resistance | Copy number | Notes |
|---|---|---|---|---|
| pZS1 | % Original repressilator plasmid | ampR | pSC101 | |
| pZS1 w/ OR2* mutation | % | ampR | pSC101 | minimize passages |
| pZE21-GFP(AAV) | % Original repressilator reporter (pTetO1) | kanR | colE1 | |
| pZE21-eGFP(ASV) | % pZE21-GFPAAV with eGFP replacement | kanR | colE1 | |
| pET21a(+)-Histag-Cerulean | Expression vector for Cerulean purification | ampR | colE1 | c. Transcriptic Inc. |
| pET21a(+)-Histag-Citrine | Expression vector for Citrine purification | ampR | colE1 | c. Transcriptic Inc. |
| pET21a(+)-Histag-mCherry | Expression vector for mCherry purification | ampR | colE1 | c. Transcriptic Inc. |
| pTetR(r)-BCD7-Citrine | % transfer fxns | kanR | pSC101* | |
| pTetR-BCD7-Citrine | # transfer fxns | kanR | pSC101* | c. Transcriptic Inc. |
| pSrpR-BCD7-Citrine | # transfer fxns | kanR | pSC101* | c. Transcriptic Inc. |
| pQacR-BCD7-Citrine | # transfer fxns, in vitro reporter | kanR | pSC101* | c. Transcriptic Inc. |
| pPhlF-BCD7-Citrine | # transfer fxns, in vitro reporter | kanR | pSC101* | c. Transcriptic Inc. |
| pLacI-BCD7-Citrine | & transfer fxns | kanR | pSC101* | c. Transcriptic Inc. |
| pLacI(r)-BCD7-Citrine | % transfer fxns | kanR | pSC101* | |
| pCI(OR2*)-BCD7-Citrine | * transfer fxns | kanR | pSC101* | |
| pCI-BCD7-Citrine | % transfer fxns | kanR | pSC101* | |
| pBetI-BCD7-Citrine | # transfer fxns | kanR | pSC101* | c. Transcriptic Inc. |
| 3n1 | oscillator plasmid | kanR | pSC101* | minimize passages |
| 3n2 | oscillator plasmid | kanR | pSC101* | minimize passages |
| 5n1 | oscillator plasmid | kanR | pSC101* | minimize passages |
| 5n2 | oscillator plasmid | kanR | pSC101* | minimize passages |
| pBetI-BCD7-phlF-ssrA(LAA) | # for building 3n1 | ampR | pSC101* | |
| pBetI-BCD7-qacR-ssrA(LAA) | # for building 5n2 | ampR | pSC101* | |
| pLacO1-BCD7-tetR-ssrA(LAA) | & for building 5n1 | ampR | colE1 | amplify in lacI repressor strain |
| pLambdaCI-BCD7-lacI-ssrA(LAA) | % for building 5n1 | ampR | colE1 | amplify in lambdaCI repressor strain |
| pPhlF-BCD7-srpR-ssrA(LAA) | # for building 3n1, 3n2, 5n1, 5n2 | ampR | pSC101* | |
| pQacR-BCD7-tetR-ssrA(LAA) | # for building 5n2 | ampR | pSC101* | |
| pSrpR-BCD7-betI-ssrA(LAA) | # for building 3n1, 5n2 | ampR | pSC101* | |
| pSrpR-BCD7-lambdaCI-ssrA(LAA) | # for building 5n1 | ampR | pSC101* | |
| pSrpR-BCD7-tetR-ssrA(LAA) | # for building 3n2 | ampR | pSC101* | |
| pTetR-BCD7-phlF-ssrA(LAA) | # for building 3n2, 5n1, 5n2 | ampR | colE1 | amplify in tetR repressor strain |

| | | | | |
|---|---|---|---|---|
| pPhlF-BCD20-sfGFP-ssrA(LAA) | # 1 color strong reporter used in study | ampR | colE1 | |
| pPhlF-BCD22-sfGFP-ssrA(LAA) | # 1 color weak reporter used in study | ampR | colE1 | |
| pPhlF-BCD20-Citrine-ssrA(LAA) | # for building 3-color reporter plasmid | ampR | colE1 | |
| pSrpR-BCD20-Cerulean-ssrA(LAA) | # for building 3-color reporter plasmid | ampR | colE1 | |
| pTetR-BCD20-mCherry-ssrA(LAA) | # for building 3-color reporter plasmid | ampR | colE1 | |
| 3-color BCD20 reporter, pPhlF/pSrpR/pJ23119-tetO | 3 color reporter plasmid | cmR | colE1 | minimize passages |
| pTetR(r)-Citrine(ASV) | % in vitro reporter | kanR | colE1 | |
| pTetR(r)-Cerulean(ASV) | % in vitro reporter | kanR | colE1 | |
| pLacI(r)-mCherry(ASV) | % in vitro reporter | kanR | colE1 | |
| pLacI(r)-Cerulean(ASV) | % in vitro reporter | kanR | colE1 | |
| pCI-Citrine-(ASV) | % in vitro reporter | kanR | colE1 | |
| pLacI(r)TetR(ASV) | % for initial conditions experiment | kanR | colE1 | |
| pTetR(r)-CI(ASV) | % for initial conditions experiment | kanR | colE1 | |

\# Promoter from Stanton et al. (2014)
$ Promoter from Anderson promoter panel
% Promoter from Elowitz and Leibler (2000)
& Promoter from Lutz and Bujard (1997)
* Promoter from Rosenfeld et al. (2005)

## C) Strains used in this study.

| Name | *E. coli* type | Resistance | Notes |
|---|---|---|---|
| Rosetta2 | JS006 | cmR | |
| pZS1 + pZE21-GFP(AAV) | JS006 | kanR ampR | |
| pET21a(+)-Histag-Citrine | BL21-DE3 | ampR | |
| pET21a(+)-Histag-Cerulean | BL21-DE3 | ampR | |
| pET21a(+)-Histag-mCherry | BL21-DE3 | ampR | |
| 3n1 + pPhlF-BCD20-sfGFP-ssrA(LAA) | JS006 | kanR ampR | minimize passages |
| 3n1 + pPhlF-BCD22-sfGFP-ssrA(LAA) | JS006 | kanR ampR | minimize passages |
| 3n2 + pPhlF-BCD20-sfGFP-ssrA(LAA) | JS006 | kanR ampR | minimize passages |
| 3n2 + pPhlF-BCD22-sfGFP-ssrA(LAA) | JS006 | kanR ampR | minimize passages |
| 5n1 + pPhlF-BCD20-sfGFP-ssrA(LAA) | JS006 | kanR ampR | cells unhealthy, minimize passages |
| 5n1 + pPhlF-BCD22-sfGFP-ssrA(LAA) | JS006 | kanR ampR | minimize passages |
| 5n2 + pPhlF-BCD20-sfGFP-ssrA(LAA) | JS006 | kanR ampR | minimize passages |
| 5n2 + pPhlF-BCD22-sfGFP-ssrA(LAA) | JS006 | kanR ampR | minimize passages |
| 3n2 + 3-color BCD20 reporter, pPhlF/pSrpR/pJ23119-tetO | JS006 | kanR cmR | minimize passages |
| pZS1 + pZE21-eGFP(ASV) | JS006 | kanR ampR | |
| pZS1 w/ OR2* mutation + pZS21-eGFP(ASV) | JS006 | kanR ampR | |

## D) DNA concentrations used in experiments.

| Experiment | DNA and concentration | Type of DNA |
|---|---|---|
| | | |
| **Repressilator orig./$O_R$2*, 3color** | Repressilator pZS1 or pZS1 w/ $O_R$2* mutation, 0.5 nM (if not otherwise indicated) | Plasmid |
| | pTetR(r)-Cerulean(ASV), 5 nM | Plasmid |
| | pLacI(r)-mCherry(ASV), 5 nM | Plasmid |
| | pCI-Citrine(ASV), 5 nM | Plasmid |
| | | |
| **Repressilator, initial conditions** | **Reaction in nano-reactor:** | |
| | Repressilator pZS1, 5 nM | Plasmid |
| | pLacI(r)-Cerulean(ASV), 5 nM | Plasmid |
| | pTetR(r)-Citrine-(ASV), 5 nM | Plasmid |
| | **Pre-synthesis reaction (CI):** | |
| | pTetR(r)-Citrine(ASV), 5 nM | Plasmid |
| | pTetR(r)-CI(ASV), 5 nM | Plasmid |
| | **Pre-synthesis reaction (TetR):** | |
| | pLacI(r)-Cerulean(ASV), 5 nM | Plasmid |
| | pLacI(r)TetR(ASV), 5 nM | Plasmid |
| | | |
| **Response curve measurements** | Promoter plasmid: pXXX-BCD7-Citrine, 1 nM | Plasmid |
| | Repressor template: pJ23151-BCD7-XXX, 0-2.5 nM | Linear |
| | Repressor reporter: pJ23151-BCD7-Cerulean, 0-2.5 nM | Linear |
| | | |
| **3n1** | 3n1 oscillator plasmid, 5 nM | Plasmid |
| | pPhlF-BCD7-Citrine, 2.5 nM | Plasmid |
| | | |
| **3n2** | pJ23119-tetO-BCD2-phlF-ssrA(LAA), 1.5 nM | Linear |
| | pPhlF-BCD2-srpR-ssrA(LAA), 12 nM | Linear |
| | pSrpR-BCD2-tetR-ssrA(LAA), 24 nM | Linear |
| | pTetR(r)-Cerulean(ASV), 5 nM | Plasmid |
| | | |
| **3n2/no-ssrA** | pJ23119-tetO-BCD2-phlF, 1.5 nM | Linear |
| | pPhlF-BCD2-srpR, 12 nM | Linear |
| | pSrpR-BCD2-tetR, 24 nM | Linear |
| | pTetR(r)-Cerulean(ASV), 5 nM | Plasmid |
| | | |
| **4n** | pJ23119-tetO-BCD2-phlF-ssrA(LAA), 0.75 nM | Linear |
| | pLacI-BCD2-tetR-ssrA(LAA), 6 nM | Linear |

| | | |
|---|---|---|
| | pPhlF-BCD2-srpR-ssrA(LAA), 6 nM | Linear |
| | pSrpR-BCD2-lacI-ssrA(LAA), 12 nM | Linear |
| | pTetR(r)-Cerulean(ASV), 2.5 nM | Plasmid |
| | pLacI(r)-mCherry(ASV), 2.5 nM | Plasmid |
| | pPhlF-BCD7-Citrine, 2.5 nM | Plasmid |
| | | |
| **5n1** | pJ23119-tetO-BCD2-phlF-ssrA(LAA), 1.1 nM | Linear |
| | pLacI-BCD2-tetR-ssrA(LAA), 16.8 nM | Linear |
| | pLambdaCI-BCD2-lacI-ssrA(LAA), 1.4 nM | Linear |
| | pPhlF-BCD2-srpR-ssrA(LAA), 5.6 nM | Linear |
| | pSprR-BCD2-lambdaCI-ssrA(LAA), 11.2 nM | Linear |
| | pCI-Citrine(ASV), 3 nM | Plasmid |
| | pTetR(r)-Cerulean(ASV), 2.5 nM | Plasmid |
| | | |
| **5n1, plasmid DNA** | 5n1 oscillator plasmid, 5 nM | Plasmid |
| | pTetR(r)-Cerulean(ASV), 5 nM | Plasmid |
| | pLacI(r)-mCherry(ASV), 5 nM | Plasmid |
| | pCI-Citrine(ASV), 5 nM | Plasmid |
| | | |
| **5n2** | pBetI-BCD7-QacR-ssrA(LAA), 1 nM | Linear |
| | pPhlF-BCD7-srpR-ssrA(LAA), 12 nM | Linear |
| | pQacR-BCD7-tetR-ssrA(LAA), 4 nM | Linear |
| | pSrpR-BCD7-BetI-ssrA(LAA), 24 nM | Linear |
| | pTetR-BCD7-phlF-ssrA(LAA), 4 nM | Linear |
| | pTetR(r)-Cerulean(ASV), 2.5 nM | Plasmid |
| | pQacR-BCD7-Citrine, 2.5 nM | Plasmid |
